# Dying comfortably in very old age with or without dementia in different care settings – a representative “older old” population study

**DOI:** 10.1186/s12877-017-0605-2

**Published:** 2017-10-05

**Authors:** Jane Fleming, Rowan Calloway, Anouk Perrels, Morag Farquhar, Stephen Barclay, Carol Brayne, on behalf of the Cambridge City over-75s Cohort (CC75C) study, Kieran Allinson, Jackie Buck, Theodore Cosco, Daniel Davies, Tom Dening, Suvi Hokkanen, Sally Hunter, Hannah Keage, Caroline Lee, Fiona Matthews, Thais Minett, Elizabeta Mukaetova-Ladinska, Graciela Muniz Terrera, Tuomo Polvikoski, Roman Romero-Ortuna, Emily Zhao

**Affiliations:** 1 Cambridge Institute of Public Health, University of Cambridge, Forvie Site, Robinson Way, Cambridge, CB2 0SR UK; 2 Department of Public Health & Primary Cambridge, University of Cambridge, Cambridge, UK; 3 North East Thames Foundation School, London, UK; 4 Faculty of Medicine, Vrije Universiteit, Amsterdam, Netherlands; 5 School of Health Sciences, University of East Anglia, Norwich, UK; 6 Primary Care Unit, Department of Public Health & Primary Cambridge, University of Cambridge, Cambridge, UK

**Keywords:** ‘Older old’ / ‘oldest old’ / ‘old old’ / ‘old* old’, ‘End of life care’ / ‘end-of-life care’, ‘Place of care’ / ‘place of death’, Comfort, ‘Symptom control’, Aged, 80 and over, Frail elderly, Palliative care, Terminal care, Symptoms, Homes for the aged, Nursing homes

## Abstract

**Background:**

Comfort is frequently ranked important for a good death. Although rising numbers of people are dying in very old age, many with dementia, little is known about symptom control for “older old” people or whether care in different settings enables them to die comfortably. This study aims to examine, in a population-representative sample, associations between factors potentially related to reported comfort during very old people’s final illness: physical and cognitive disability, place of care and transitions in their final illness, and place of death.

**Methods:**

Retrospective analyses linked three data sources for *n* = 180 deceased study participants (68% women) aged 79–107 in a representative population-based UK study, the Cambridge City over-75s Cohort (CC75C):

i) prospective in-vivo dementia diagnoses and cognitive assessments,

ii) certified place of death records,

iii) data from interviews with relatives/close carers including symptoms and “How comfortable was he/she in his/her final illness?”

**Results:**

In the last year of life 83% were disabled in basic activities, 37% had moderate/severe dementia and 45% minimal/mild dementia or cognitive impairment. Regardless of dementia/cognitive status, three-quarters died following a final illness lasting a week or longer. 37%, 44%, 13% and 7% of the deceased were described as having been “very comfortable”, “comfortable”, “fairly comfortable” or “uncomfortable” respectively during their final illness, but reported symptoms were common: distress, pain, depression and delirium or confusion each affected 40–50%. For only 10% were no symptoms reported. There were ≥4-fold increased odds of dying comfortably associated with being in a care home during the final illness, dying in a care home, and with staying in place (dying at what death certificates record as “usual address”), whether home or care home, compared with hospital, but no significant association with disability or dementia/cognitive status, regardless of adjustment.

**Conclusions:**

These findings are consistent with reports that care homes can provide care akin to hospice for the very old and support an approach of supporting residents to stay in their care home or own home if possible. Findings on reported high prevalence of multiple symptoms can inform policy and training to improve older old people’s end-of-life care in all settings.

**Electronic supplementary material:**

The online version of this article (10.1186/s12877-017-0605-2) contains supplementary material, which is available to authorized users.

## Background

Increasing longevity means more people are dying at increasingly older ages, often with multiple co-morbidities complicating their end-of-life care [1, 2]. In the UK, for example, over just the last quarter century the proportion of deaths occurring at the age of 85 or older has risen steeply from around a fifth in 1990 to almost half current annual deaths [3]. Dementia prevalence rises with proximity to death, over and above age-associated increases [4]. Multiple symptoms are common amongst older people with dementia as they approach the end of life and poor symptom control may increase distress and worsen quality of life [5]. Past research in both hospital and long-term care settings has highlighted concerns that failure to recognise dementia as a terminal condition and communication breakdown may both contribute to poor symptom management [5, 6]. Variation in levels of symptom control for all older people dying in different care settings, with or without dementia, is an important concern. [7, 8] Comfort and effective symptom management feature high in patients’ [9–13], relatives’ [14, 15] and professionals’ [16] perceptions concerning what factors are important for a good death. In a study of advance care planning amongst older adults 92% of patients prioritised comfort [17].

Most people taking part in surveys about their future wishes [18–20] or reporting what they believe their relatives wanted [7, 21] state a preference to die at home, if expressed at all. These widely quoted findings may not be representative [22], nor reflect how views can vary with proximity to death [20, 23, 24] and personal circumstances [25, 26]. Although policy encourages support for patients to die wherever they would prefer [27, 28], dying at home is a more measurable outcome than death in preferred place of care so is often taken as a surrogate indicator of end-of-life care supporting choice [29, 30]. However, a minority of the oldest old people die in their own homes [8, 31–33] and indeed research shows that many older people anticipate preferring to be cared for elsewhere in the final illness [18, 19, 25, 26, 34]. In the UK fewer older people die in hospices or receive specialist palliative care at home than younger age-groups [35–38] and the trend for older deaths is gradually moving away from death in hospital towards long-term care facilities [39, 40].

For older people in many countries care transitions are common during the last weeks [41], months [42, 43] or year [31] of life. Those with cognitive impairment are most likely to live in and subsequently die in care homes [44, 45], while those with no or mild cognitive impairment are more likely to live at home in the community and then be admitted into acute facilities during the terminal phase [32]. As well as cognitive status, other factors have been reported to influence transitions such as gender, affluence of area and diagnosis [39, 46, 47]. In studies that analysed causes of transitions a common theme was that uncertain diagnosis, less skilled members of staff, communication difficulties and needing to access out of hours services made hospital admission more likely [43, 48–50].

Transitions towards the end of life can be burdensome and may not be associated with symptom improvement nor with better quality end-of-life care. They may result in exclusion of the main carer and a disjointed experience of the health service [51], even leading to adverse events in drug prescription [52]. Admission to hospital at the end of life is also associated with greater cost [53–55]: UK health service spending exceeding £750 m a year on emergency admissions ending in death [27], and evidence suggests hospitalisation for patients with severe dementia is not cost effective [56].

There are several gaps in the literature concerning comfort at the end of life amongst older old people. Although there is extensive research concerning their preferred place of care, few studies have compared end-of-life care across different settings and there is little evidence to support the commonly-held view that transitions have a negative effect on quality of end-of-life care. Few studies have been able to link individual-level data including dementia or cognitive status prospectively assessed before death with information on where and how dying individuals were cared for. This study has achieved this, examining in a population-representative sample the associations between factors potentially related to reported comfort during the final illness of very old people – in particular, their cognitive and physical disability, the setting where they were cared for in their final illness, and where they died.

## Methods

Analyses of data from a prospective population-based study of ageing in which almost all respondents have died, the Cambridge City over-75s Cohort (CC75C) study [57], linked i) in vivo dementia diagnoses and cognitive assessments with ii) certified place of death records and iii) data from interviews with bereaved relatives or other closely involved carers (“informants”) for the maximum sub-sample with all three data sources (*n* = 180). The CC75C study [58] has followed up 2166 men and women aged at least 75 at baseline (1985–87) enrolled through general practices (95% response rate) in Cambridge, UK, with surveys every few years until death (all deceased by December 2015). The study remained highly representative of the older old population, with mortality the main reason for attrition.

### Cognitive assessment

Each survey wave has included a detailed cognitive assessment, including the Mini Mental State Examination (MMSE) [59]. Almost half the cohort (*n* = 1022) had further in-depth investigation at least once following Surveys 1–3: the Cambridge Mental Disorders of the Elderly Examination (CAMDEX) [60], a psychiatrist-administered interview schedule for the diagnosis of dementia.

After death, a consensus diagnosis of dementia status at death consistent with the Diagnostic and Statistical Manual of Mental Disorders version 4 (DSM-IV) [61] criteria was made by two clinicians experienced in old age psychiatry. This diagnosis was based on review of all available information including survey data, proxy informant data, general practitioner (GP) confirmation of dementia diagnosis, death certificates and data from retrospective informant interviews after participants had died [62].

### Definition of dementia status

“Dementia status” was categorized in three categories: “no cognitive impairment – no dementia”, “cognitive impairment - minimal/mild dementia” and “moderate/severe dementia”. Participants who had undergone CAMDEX assessments less than a year before death or clinical diagnosis at death were assigned to these categories according to dementia severity ratings. Those whose death certificate recorded dementia were assumed to have moderate/severe dementia. Participants who had no confirmed dementia status were categorized on the basis of severity of cognitive impairment as measured by MMSE less than two years before death: no dementia – no cognitive impairment (26–30), cognitive impairment – minimal/mild dementia (18–25) and moderate/severe dementia (0–17).

### Reported symptoms and comfort

For participants who joined the study’s brain donor programme, or another sub-study, a retrospective interview after their death with a close relative or carer collected information on the period between the participant’s last interview and death (*n* = 290). Funding limitations meant only informants for these participants were approached (81% response rate) and for a minority approaches to request interview were missed but followed-up later (*n* = 10 more than ten years after the participant died). This included the CAMDEX informant interview schedule and questions about the participant’s care at the end of their life [63]. Questions included “Overall, how comfortable was s/he during the final illness?” (very/fairly comfortable, somewhat/very uncomfortable), “Did s/he suffer any of the following symptoms during his/her final illness?” (see Results for symptom list) and “If YES to any of these, was treatment given for this problem and how effective was it?” (free text responses categorised “Yes”, “Somewhat”, “No” or “Unknown”). Informants were asked for their estimate of duration of what they considered the “final illness” (≤1 week, 1 week to 1 month, >1 month).

### Analyses

The analysed sample (*n* = 180) was all participants with retrospective informant interviews providing data on comfort in their final illness, excluding participants of unknown dementia status whose last cognitive assessment was two or more years before they died. Descriptive analyses summarised participant and informant characteristics, the prevalence of reported symptoms, treatment and effectiveness for these symptoms, and reported comfort in the final illness. Logistic regression examined the relationship between being described as comfortable (four responses dichotomised comfortable/uncomfortable) in the final illness and each potentially related factor. Factors identified as significantly associated with comfort in univariate analyses (*p* < 0.05) and dementia status, which was hypothesised a priori to be a relevant factor, were separately entered in regression analyses adjusting for each of these factors individually. The extent of correlations between factors found to be significant in univariate regression, and between these and dementia status, were explored using descriptive bivariate cross-tabulations. Sample size, the number of factors of interest and their strong inter-dependence meant multiple regression was inappropriate. All statistical and descriptive analyses were performed using the statistical package STATA Version 12 (Stata Corporations, College Station, TX).

### Patient involvement

When the CC75C study began in the mid-1980s there was no patient involvement. Participants and their relatives have been included in dissemination of the cohort study’s findings through annual newsletters until the last participant died in 2015. The broad public involvement in the James Lind Alliance Palliative and End of Life Care Priority Setting Partnership [64] helped inform research questions for the study here reported.

### Ethical approval

Each stage of the CC75C study has been approved by the local Research Ethics Committee.

## Results

### Characteristics of the study participants

**Fig. 1:**
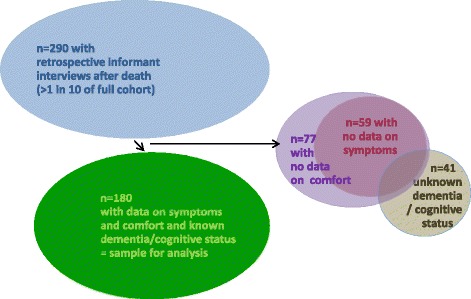
Identification of participants with retrospective informant interviews for analysis sample. This study, nested in a representative population-based cohort, analysed data from the branch study in which for approximately one in ten of the full cohort, by design, proxy informant interviews were conducted after participants died. Participants were excluded if their dementia / cognitive status by the time they died was unknown or if retrospective informant interview questions on their level of comfort and symptoms experienced during their final illness had missing data

The sample for this analysis was all 180 cohort participants (57 men, 123 women) for whom dementia status was defined and informant data were available reporting symptoms and how comfortable they were in their final illness (see Fig. 1). Table 1 shows the deceased study participants’ characteristics, overall and by dementia status.

**Table 1:** Characteristics of deceased study participants

	No dementia, no cognitive impairment *n* = 33				Minimal-mild dementia, moderate cognitive impairment *n* = 81				Moderate-severe dementia, severe cognitive impairment *n* = 66				Total *n* = 180			
	n		(%)		n		(%)		n		(%)		n		(%)	
Age at death																
Median [IQR]	89.7		[87.1–92.0]		90.7		[86.9–94.2]		92.3		[88.5–95.7]		91.3		[87.5–94.4]	
[range]			[81.6–102.8]				[79.2–106.7]				[89.9–100.8]				[79.2–106.7]	
≤ 89 years old	19		(58)		36		(44)		23		(35)		78		(43)	
≥ 90 years old	14		(42)		45		(56)		43		(65)		102		(57)	
Sex																
Male	16		(48)		27		(33)		14		(21)		57		(32)	
Female	17		(52)		54		(67)		52		(79)		123		(68)	
Marital status																
Married	5		(15)		16		(20)		17		(26)		38		(21)	
Widowed	27		(82)		54		(67)		42		(64)		123		(68)	
Separated/Divorced	1		(3)		0		(0)		1		(2)		2		(1)	
Single	0		(0)		11		(14)		6		(9)		17		(9)	
Education																
Left school aged ≤14	17		(52)		56		(69)		42		(64)		115		(64)	
Left school aged ≥15	16		(48)		25		(31)		23		(35)		64		(36)	
School leaving age unknown	0		(0)		0		(0)		1		(2)		1		(<1)	
Social class^a^																
Non-manual	17		(52)		30		(37)		32		(48)		79		(44)	
Manual	16		(48)		51		(63)		32		(48)		99		(55)	
Social class unknown	0		(0)		0		(0)		2		(3)		2		(1)	
Place of residence last interview^b^																
Home (house, flat or ‘granny flat’)	31		(94)		73		(90)		30		(45)		134		(74)	
alone	20		(65)		48		(66)		14		(47)		82		(61)	
with others	11		(35)		25		(34)		16		(53)		52		(39)	
Long-term care^a^	2		(6)		8		(10)		36		(55)		46		(26)	
residential care home	2		(100)		7		(88)		33		(92)		42		(92)	
nursing home	0		(0)		0		(0)		2		(6)		2		(4)	
long-stay ward	0		(0)		1		(13)		1		(3)		2		(4)	
Disability in activities of daily living when last interviewed																
No disability	7		(21)		2		(2)		1		(2)		10		(6)	
Disability: instrumental ADLs only	5		(15)		14		(17)		1		(2)		20		(11)	
Disability: basic + instrumental ADLs	21		(64)		65		(80)		64		(97)		150		(83)	
Receiving service support (if community-dwelling)																
None	10		(32)		21		(29)		10		(33)		41		(31)	
Once a week	7		(23)		21		(29)		10		(33)		38		(28)	
More than once	14		(45)		29		(40)		8		(27)		51		(38)	
Unknown	0		(0)		2		(3)		2		(7)		4		(3)	
Hospital admissions between last interview and death																
None	17		(52)		42		(52)		39		(59)		98		(54)	
One or more	15		(45)		35		(43)		25		(38)		75		(42)	
Unknown	1		(3)		4		(5)		2		(3)		7		(4)	
Hospital admissions per year between last interview and death, if any																
Median [IQR]	0.9		[0.6–1.7]		0.7		[0.5–1.7]		0.6		[0.4–1.2]		0.8		[0.5–1.6]	
[range]			[(0.2–29.6]				[0.2–3.7]				[0.2–4.6]				[0.2–29.6]	
Duration of final illness																
Less than 1 week	8		(24)		19		(23)		17		(26)		44		(24)	
7 days up to 1 month	16		(49)		36		(44)		28		(42)		80		(44)	
1 month or more	9		(27)		26		(32)		21		(32)		56		(32)	
Place of care during final illness^b^																
Home	11		(33)		17		(21)		5		(8)		33		(18)	
Long-term care	4		(12)		18		(22)		50		(76)		72		(40)	
Hospital	18		(55)		46		(57)		11		(17)		75		(42)	
Place of death^b^																
Home	4		(12)		12		(15)		3		(5)		19		(11)	
Long-term care	7		(21)		19		(23)		50		(76)		76		(42)	
Hospital	22		(67)		50		(62)		13		(20)		85		(47)	
Place of death not usual address																
No	7		(21)		25		(31)		43		(65)		75		(42)	
Yes	26		(79)		56		(69)		23		(35)		105		(58)	
Time from last interview to death																
Years: median [IQR]	1.8		[1.2–2.5]		1.6		[0.8–2.5]		2.2		[1.0–3.3]		1.8		[1.0–2.8]	
[range]			[0.1–5.3]				[0.3–5.7]				[0.1–6.8]				[0.1–6.8]	

Footnotes

Column percentages total 100% - apparent slight discrepancies due to rounding

^a^Social class categorised following contemporary UK Office of National Statistics grading of occupation reported at baseline interview:

Non-manual = I, II or IIIa, Manual = IIIb, IV or V

^b^Home: community-dwelling in a house, flat, ‘granny flat’ (part of a relative’s home) or sheltered accommodation

Long-term care: in an older people’s residential home, nursing home or long-stay ward

Hospital: in an acute hospital

**Characteristics of the deceased, shown separately for study participants of different cognitive status and overall, summarise data drawn from interviews with the participants before they died and proxy informants after the participant died, CC75C study administrative data and death certificates**

Over a third of individuals (37%) were assessed as having “moderate/severe dementia”, 45% as having “cognitive impairment – minimal/mild dementia” and 18% as having “no dementia - no cognitive impairment” when last interviewed. The age at time of death across all groups ranged from 79 to 107 years, (median [IQR] 91.3 [87.5–94.4]). Participants without dementia or cognitive impairment were younger at death than those with moderate/severe dementia (median [IQR] 89.7 [87.1–92.0], 92.3 [88.5–95.7] respectively). There were more women than men (68%, 32% respectively), especially amongst the individuals with any severity of dementia and/or cognitive impairment, and two-thirds had been widowed (68%). Demographics and distributions of cognition and dementia status in the analysis sample reflected those in the full cohort.

Participants with ‘no cognitive impairment – no dementia’ or with “cognitive impairment – minimal/mild dementia” were mainly living at home when last interviewed (94%, 90% respectively), compared with only 45% those with moderate/severe dementia. The majority of community-dwelling participants at home lived alone (61%) and received service support at least once a week when last interviewed (66%). Nearly all the participants had disabilities in both instrumental and basic activities in daily living (83%), ranging from 64% to 97% across categories of increasing dementia severity. For about three-quarters of the participants the duration of their final illness was reported to be longer than six days, regardless of dementia status.

There were marked differences in the older adults’ place of care during their final illness and their place of death depending on their dementia status. Although fewer than half the participants had been admitted to a hospital since they were last interviewed, hospital was the most common place of end-of-life care and death for individuals in the unimpaired or intermediate groups. For those with “moderate/severe dementia” long-term care was the most likely setting for both end-of-life care (76%) and death (76%), although about one in six had received hospital care during their final illness and a fifth had died in hospital. Very few of this group had received community end-of-life care or died at home (8% and 5% respectively).

Overall, the majority had experienced a change in residence or care in the period prior to death (the “usual address” recorded on their death certificate differed from their place of death), though this was far less common for people with moderate/severe dementia (35%) than for the cognitively unimpaired (79%).

The median time from the last study interview until a participant’s death was 1.8 years (IQR 1–2.8), with a slightly longer duration amongst participants with “moderate/severe dementia”.

The analysis sample broadly reflected the full representative cohort’s demographics, with very similar distributions by sex, education level and social class, but they were just over a year older at death (91.1 versus 89.9 years), more likely to be widowed (68% versus 56%) and more likely living in care (26% versus 16%). Disability levels were higher in our sample but the distribution of known dementia status and cognition were similar.

### Characteristics of the retrospective interview informants

Most informants were relatives of the participants (86%, of whom nearly two-thirds were sons or daughters) and 68% were women (see Table 2). Most had been in regular contact with the participants, seeing them more than once a week (84%). For 17% of participants who died in long-term care the only available informant was a member of staff in the care home. The median time between the participant’s death and the informant interview was 2.3 years (IQR 1.3–3.7 years; range 1 month to 14.1 years).

**Table 2:** Characteristics of informants

	Total *n* = 180	
	n	(%)
Sex		
Female	123	(68)
Male	57	(32)
Relationship to deceased		
Husband or wife	18	(10)
Son or daughter	97	(54)
Other relative	40	(22)
Friend	8	(4)
Matron or warden^a^	15	(8)
Unknown	2	(1)
Frequency of contact with deceased study participant before they died		
Lived with her/him	29	(16)
Daily	45	(25)
At least once a week	77	(43)
Less than once a week	29	(16)
Interval from study participant’s death to retrospective informant interview		
Years: median [IQR]	2.3	[1.3–3.7]

Footnotes

Column percentages total 100% for each sub-group - apparent slight discrepancies due to rounding

^a^Matron or other member of staff in a care home, or warden in a sheltered housing scheme

**Most informants were women, usually relatives, most often a daughter or son, and the majority had been in close contact with the deceased participant before they died: 84% were either living with them, visiting daily or seeing them more than once a week**

### Reported symptoms in the final illness

**Fig. 2:**
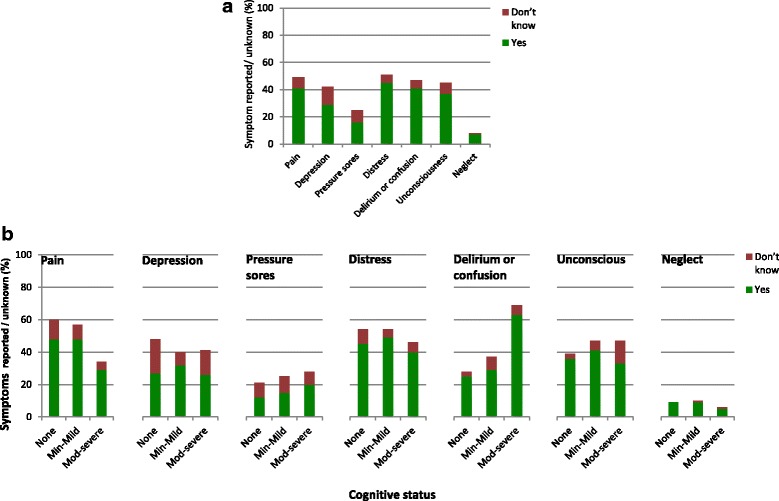
**a** Symptoms in the final illness reported by informants. Informants commonly reported that their relative had suffered at least one symptom during their final illness: distress, pain, depression and delirium or confusion were each reported to affect 40–50% of the deceased study participants. Depending on the symptom, 6–13% of informants were unsure whether or not their relative had experienced symptoms. **b** Symptoms in the final illness reported by informants for older people of different cognitive status. Pain was less commonly reported to have affected the most cognitively impaired individuals than those with milder dementia/cognitive impairment or none (29% versus 48%), a difference just touching statistical significance (*p* = 0.05). There were no other significant differences between cognitive groups in reported symptoms during the final illness except for the expected higher prevalence of ‘delirium or confusion’ amongst those most cognitively impaired

**Fig. 3:**
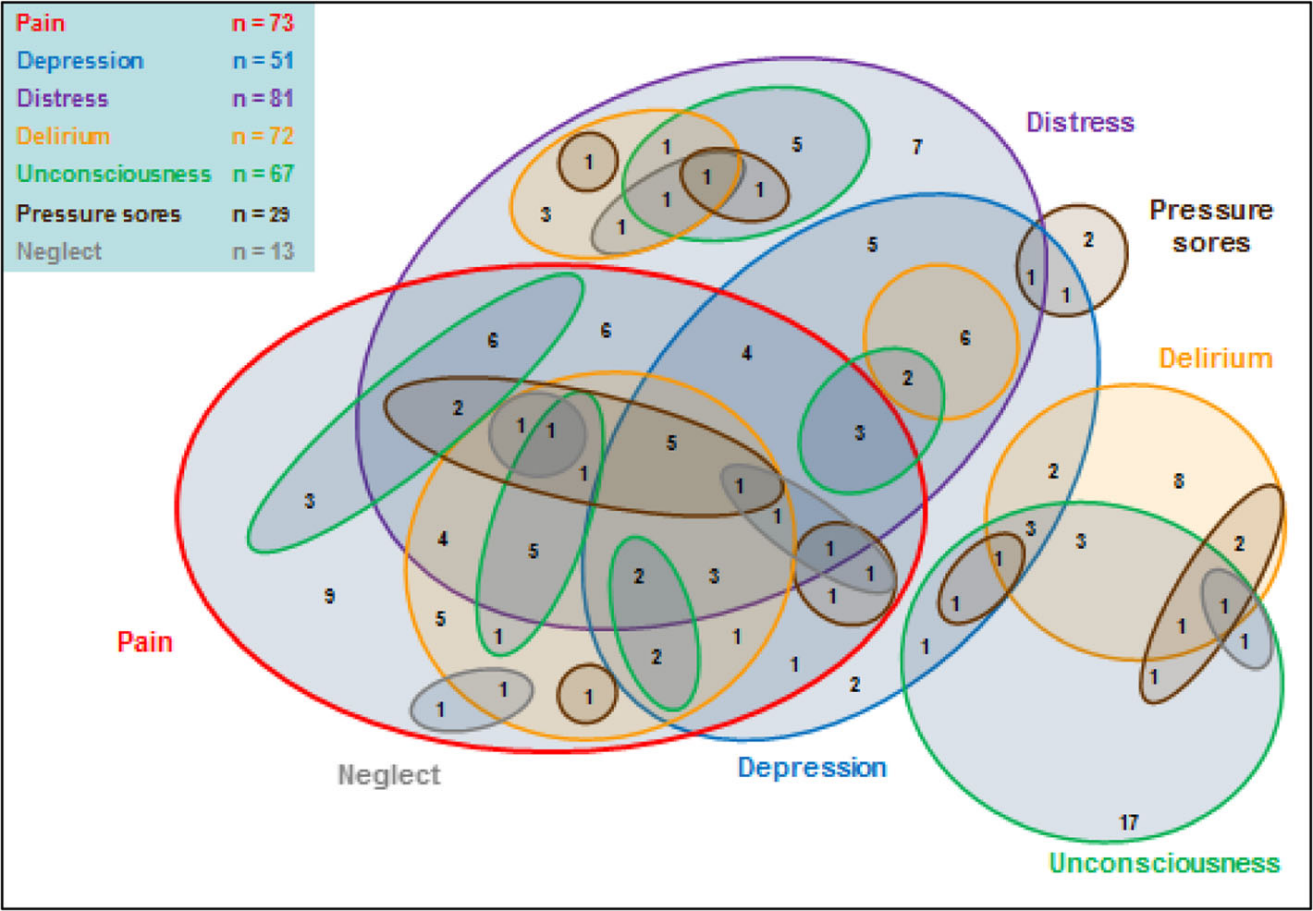
Distribution of symptoms in the final illness Informants’ reports describe a complex overlap of multiple symptoms experienced by very old people in their final illness. Illustrates this for participants with any reported symptoms (*n* = 155 with for whom informants replied “Yes” for at least one symptom; *n* = 18 with only “No” responses and *n* = 7 with only “No” or “Don’t know” responses not shown). The majority (61%) of participants were reported to have suffered from at least two of the seven symptoms that the study interviews asked about. For only 10% were none of these symptoms reported, and for a further 4% informants were unsure whether they had suffered any of these symptoms

Informants were asked whether the study participant suffered any of a list of seven symptoms in their final illness. The distribution of symptoms reported (Fig. 2a **)** varied for older people of different cognitive status (Fig. 2b **)**; both figures show firm reports and cases when informants were unsure whether or not a symptom was experienced (responses “Yes” and “Don’t know”). Apart from expected high rates of “delirium and confusion” amongst those with moderate/severe dementia, pain and distress were the most commonly reported symptoms. Informants were most unsure whether or not the participant had suffered from depression. For only *n* = 18 (10%) were none of these symptoms reported, and for a further *n* = 7 (4%) informants were unsure whether the participant had suffered any of these symptoms. A quarter were reported to have experienced just one symptom, but the majority (61%) reportedly suffered at least two symptoms and 42% three or more. Figure 3 illustrates for the full sample the complex overlap of symptoms, showing only symptoms reported as “Yes”.

**Fig. 4:**
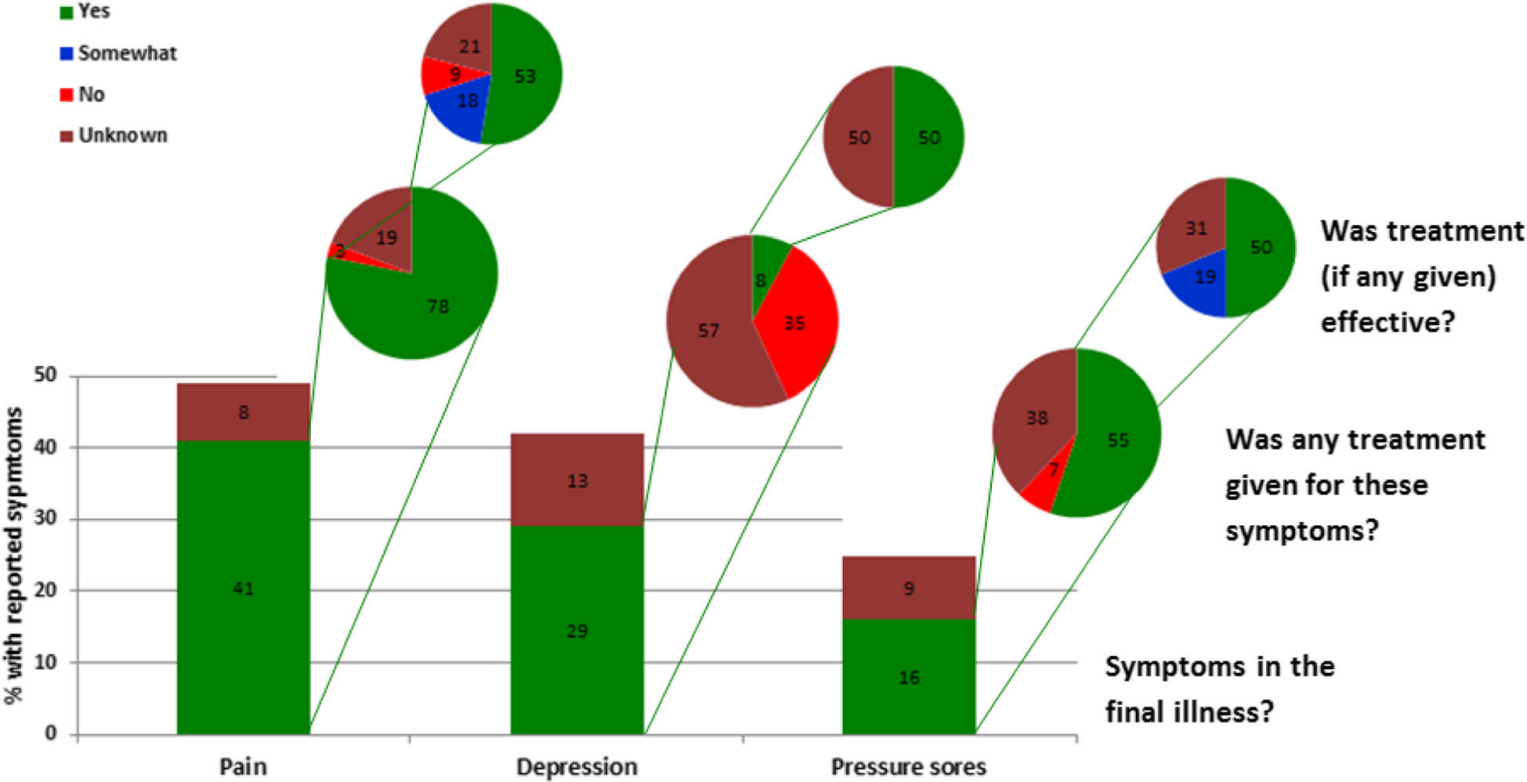
Treatment reported for pain, depression and pressure sores and reported effectiveness. Illustrates what proportion of three treatable symptoms reported to have affected deceased participants in their final illness – pain, depression and pressure sores – were said by informants to have been given any treatment and, if so, what proportion of those treated were reported to have had effective symptom relief. For example, of the 29% reported as suffering depression only 8% received treatment, of which only half were reported to have been effective

Figure 4 illustrates what percentage of the people who were reported to have had pain (*n* = 73, 41%), depression (*n* = 51, 29%) and pressure sores (*n* = 29, 16%) were also reported to have had treatment for these and, of these treated, for what percentage treatment was reported to have been effective. For example, of the 41% reported to have had pain 78% reportedly had pain-relieving treatment, for whom informants reported 53% found this effective, i.e. only 41% of those in pain were thought to have had adequate pain relief (53% of 78%). Informants were frequently uncertain as to whether these symptoms were experienced, treated and, if so, whether symptoms were relieved. This was particularly so for depression and pressure sores.

### Reported comfort in the final illness

“How comfortable was s/he in his/her final illness” was answered positively by the majority of informants: 37% described the deceased older person as having been “very comfortable”, 44% “comfortable”, 13% “somewhat uncomfortable” and 7% “very uncomfortable”. These high proportions reported as comfortable included many with symptoms as described above, with informants reporting effective treatment more often for those rated comfortable than for those rated uncomfortable (60% versus 44%, non-significant difference, *p* = 0.2, in the small sub-sample reported to have received symptom treatment: *n* = 67). Informants for individuals in the more severe cognitive impairment group were more likely uncertain whether any treatment given was effective than for the cognitively intact (25% and 8% respectively, *p* = 0.6) and less likely to report treatment as effective (52% and 75% respectively, *p* = 0.3). Nonetheless, the most cognitively impaired individuals were most likely to be rated comfortable (respectively 76%, 75% and 89% of each dementia status category, *p* = 0.07), a finding consistent with the high prevalence of reported comfort for those whose end-of-life care was in long-term care (see Table 3).

**Table 3:** Factors potentially related to reported comfort during the final illness

	n “comfortable” / total each group (%)	Unadjusted OR (95% C.I.)
Socio-Demographics		
Age	145/180	
< 90 years old	62/78 (79)	1
≥ 90 years old	83/102 (81)	1.1 (0.5–2.4)
Sex	145/180	
Male	46/57 (81)	1
Female	99/123 (80)	1.0 (0.4–2.2)
Marital status^a^	143/178	
Married	31/38 (82)	1
Widowed	98/123 (80)	0.9 (0.3–2.2)
Single	14/17 (82)	1.1 (0.2–4.7)
School leaving age^a^	144/179	
≤ 14 years old	90/115 (78)	1
≥ 15 years old	54/64 (84)	1.5 (0.7–3.4)
Social class^ab^	143/178	
Non-manual	61/79 (77)	1
Manual	82/99 (83)	1.4 (0.7–3.0)
Place of residence at last interview^c^	145/180	
Home	104/134 (78)	1
Long-term care	41/46 (89)	2.4 (0.9–6.5)
Location of End of Life Care		
Place of care during final illness^c^	145/180	
Hospital	55/75 (73)	1
Home	24/33 (73)	1.0 (0.4–2.4)
Long-term care	66/72 (92)	**4.0 (1.5–10.7)**
Place of death^c^	145/180	
Hospital	59/85 (69)	1
Home	17/19 (89)	3.7 (0.8–17.4)
Long-term care	69/76 (91)	**4.3 (1.8–10.7)**
Places of care and death - transitions at the end of life^ad^	137/169	
Care in final illness: hospital Place of death: hospital	53/71 (75)	1
Final illness: home Place of death: hospital	6/13 (46)	***0.3 (0.1–1.0)***
Final illness: home Place of death: home	14/16 (88)	2.4 (0.5–11.5)
Final illness: long-term care Place of death: long-term care	64/69 (93)	**4.3 (1.5–12.5)**
Place of death same as usual address	145/180	
No	76/105 (72)	1
Yes	69/75 (92)	**4.4 (1.7–11.2)**
Health & Disability		
Dementia status	145/180	
No dementia-cognitively intact	25/33 (76)	1
Cognitively impaired +/− minimal/mild dem	61/81 (75)	1.0 (0.4–2.5)
Moderate/severe dementia	59/66 (89)	2.7 (0.9–8.2)
Duration of final illness^a^	145/180	
Less than a week	39/44 (89)	1
7 days up to a month	62/80 (78)	0.4 (0.2–1.3)
1 month or more	44/56 (79)	0.5 (0.2–1.5)
No. of hospital admissions/year since last interview^a^	138/173	
None	79/98 (81)	1
One or more	59/75 (79)	0.9 (0.4–1.9)
Functional disabilities in ADLs	145/180	
No basic or instrumental ADL disability	8/10 (80)	1
Instrumental ADL disability only	14/ 20 (70)	0.6 (0.1–3.6)
Basic + instrumental ADL disability	123/150 (82)	1.1 (0.2–5.7)
Receiving service support (*excluding long-term care*)^a^	100/130	
More than once a week	33/51 (65)	1
Once a week	32/38 (84)	***2.9 (1.0–8.3)***
None	35/41 (85)	**3.2 (1.1–9.0)**
Informants & Interviews		
Informant’s sex	145/180	
Male	50/57 (88)	1
Female	95/123 (77)	0.5 (0.2–1.2)
Informant’s relationship to participant^a^	143/178	
Husband or wife	15/18 (83)	1
Son or daughter	78/97 (80)	0.8 (0.2–3.1)
Other relative	8/12 (67)	0.4 (0.1–2.2)
Friend	6/8 (75)	0.6 (0.1–4.5)
Warden or matron	13/15 (87)	1.3 (0.2–9.0)
Other	23/28 (82)	0.9 (0.2–4.4)
How often informant saw participant	145/180	
Lived with participant	24/29 (83)	1
Daily	35/45 (78)	0.7 (0.2–2.4)
More than once a week	45/55 (82)	0.9 (0.3–3.1)
Once a week	18/22 (82)	0.9 (0.2–4.0)
Less than once a week	23/29 (79)	0.8 (0.2–3.0)
Interval from last survey interview to death	145/180	
< median	73/90 (81)	1
≥ median	72/90 (80)	0.9 (0.4–1.9)
Interval from death to informant interview	145/180	
< median	70/90 (78)	1
≥ median	75/90 (83)	1.4 (0.7–3.0)

Footnotes

OR = Odds Ratio (shown to 1 decimal point)

**bold OR** = significant (*p* < 0.05); ***italic bold OR*** = borderline significant (*p* = 0.5)

95% C.I. = 95% Confidence Interval (shown to 1 decimal point)

ADL(s) = Activity (*or* Activities) of Daily Living

^a^Variables in which categories total < 180 had missing data

^b^Social class categorised following contemporary UK Office of National Statistics grading of occupation reported at baseline interview: Non-manual = I, II or IIIa, Manual = IIIb, IV or V

^c^Home: community-dwelling in a house, flat, ‘granny flat’ (part of a relative’s home) or sheltered accommodation

Long-term care: in an older people’s residential home, nursing home or long-stay ward

^d^To explore the effects of transitions in place of care at the very end of life, the variable *Places of care at the end of life* was derived from data on where each individual was cared for during their final illness and where they died. Frequencies in other categories were too small (between 1 and 4 people) to calculate any estimates for other combinations of place of care

**End-of-life care in long-term care, avoiding transition between care settings at the end of life and not needing support from formal services were associated with dying comfortably, as reported by informants**

Table 3 shows the distribution of reported comfort in the final illness across categories of each descriptive characteristic and gives the unadjusted odds ratio (OR) for reportedly having been comfortable associated with each factor. Compared with care or death in hospital, there was at least a 4-fold increased odds of dying comfortably associated with being in a care home during the final illness, with dying in a care home, with staying in place in a care home (i.e. when a care home was the location for both end-of-life care and death) and dying at the participant’s “usual address” (home or care home). Although people who died at home or in long-term care were similarly commonly reported comfortable (89% and 91%, versus 69% for hospital deaths), there were too few deaths at home for the association between dying at home and comfort to reach significance: OR 3.7 (95% CI 0.8–17.4). People with home or hospital care in the final illness were equally commonly reported comfortable (both 73%, versus 92% in long-term care). However, compared with those who were cared for in hospital until death, the odds of dying comfortably were just significantly lower for those who died in hospital after having been cared for at home: OR 0.29 (95% CI 0.09–0.98). Those who were living at home when last surveyed, needing no support from formal services or needing support only once a week, had a 3-fold increased odds of a comfortable death compared with those needing support twice a week or more.

Adjusting for the covariates found significant in univariate analyses had minimal impact on most associations with reported comfort, but reduced effect sizes and significance for some most closely inter-related factors, though not all (see Additional file 1: Table S1). For example, taking into account ‘death at usual address’ reduced the effect of end-of-life care location factors, but adjusting for the level of service support needed in the community-dwelling sub-sample strengthened the association with comfort of dying in long-term care. The non-significant association between dementia status and dying comfortably was slightly reduced by adjusting for any significant factor. Adjusting for the time interval between participant deaths and informant interviews also made minimal or no difference to associations with other factors (see Additional file 2: Table S2).

**Fig. 5:**
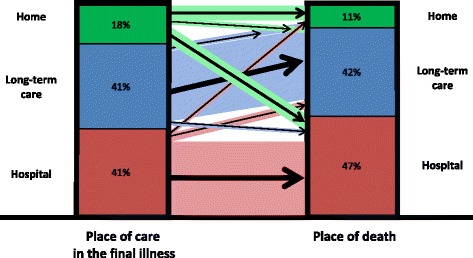
Transitions between place of care in the final illness and place of death. More than half the individuals whose care during the final illness was at home died elsewhere, whereas the majority of those whose end-of-life care was in hospital or long-term care died there

Figure 5 illustrates how closely inter-related the place of care factors are: the majority of people who died in a care home had also been cared for in their final illness in a care home. This was usually the same home. Likewise, when care during the final illness was in hospital, this was usually the place of death too. However, more than half the individuals whose end-of-life care was at home died elsewhere (12% in care homes, 39% in hospitals), and this tended to be most likely for those who had needed more formal service support than less (79%, 25%, 36% for ≥2, 1, 0 service contacts per week respectively, *p* = 0.03).

## Discussion

Death not occurring until the tenth decade or later is becoming increasingly common, but dying in advanced old age is still relatively rarely described. This population study provides much-needed evidence to inform service planning to support very old people to die comfortably. Nearly half this representative sample of very old people, who died at a median age of 91, experienced at least three symptoms during their final illness. Pain and distress were the most commonly reported, but no clear pattern of symptom clusters emerged. There was some uncertainty about which symptoms were present, particularly depression. Even the largest single symptom group, unconsciousness, was small, suggesting fewer than 10% of the very old simply ‘slip away’. Of the treatable symptoms reported, pain was addressed in the majority affected, though effectively for only half these, but only a fraction of those with depression were treated. Nonetheless, despite high symptom prevalence when dying, death was described as comfortable for the majority. The association found between comfort and effective treatment, non-significant within the small sub-sample reported to have had symptoms addressed, may partially explain informants describing many with multiple symptoms as comfortable. Comfort was significantly associated with being cared for in a long term care facility, dying in a long term care facility and also with dying at the individual’s usual address. Despite the high proportions of the most cognitively impaired in all these categories, the association between dementia status and reported comfort at the end of life was not significant. Of the majority still living at home when last interviewed, those receiving support more than once a week were more likely to move before they died and less likely to be described as dying comfortably.

A major strength of this study is its combination of prospectively collected data from a representative population-based cohort with retrospective informant interviews and death certificate data, allowing individual-level examination of aspects of end-of-life care experienced by very old people. The CC75C study is one of the very few longstanding population cohorts with participants of such advanced age followed-up to death and data including cognitive assessments from the last year of life. Our sample is sizeable for end-of-life research with very old people, but our focus on deaths in advanced old age inevitably means it is nonetheless too small to fully examine all potentially relevant factors. The hypotheses generated by some of these findings may need testing by combining studies in order to gain sufficient power in future research.

The very nature of such longitudinal research also carries limitations, such as the inevitable absence of more recent measurement approaches [65] and the extended data-collection period. It is possible that experience of death is changing over time, though one study that explored this (in a younger old age-range) found minimal 10-year change in home-to-hospital end-of-life transfers and proportions of deaths at home [66]. Information explaining reasons for hospitalisation before death is limited. However, few studies have been able to look at a whole “older old” population and the CC75C data provide rare insight. Limitations of proxy data from bereaved relatives are well-recognised [67, 68] and our informant interview post-bereavement timings varied. Others have explored stability of family informants’ responses over time [69, 70] and our analyses found no impact on findings of these interval variations. A further limitation with potentially more relevance to our findings is the fact that, although drawn from a representative population-based cohort, our sample inevitably did not include those for whom there was no informant to give a retrospective interview. Assessing potential missing data impacts, we confirmed the analysis sample broadly reflected the full representative cohort.

Other large population-based studies find prevalences of dementia and cognitive impairment at the end of life at advanced age in line with this study, though most reporting advanced age prevalence [71–74] are not specifically from populations near death [2, 4]. As expected, we found higher disability levels than reported from other “older old” but not end-of-life cohorts [75, 76] and from studies of older people nearing death but including a younger old age range [77, 78]. Other population-wide research provides broadly similar figures for the proportion of those dying in different settings, with general concordance that those with no cognitive impairment were most likely to live at home and then move to hospital whereas those with moderate-severe dementia were most likely to live and die in a care home [39, 44, 47, 66, 79]. Other UK research confirms transitions to hospital before death are usually from home and least likely from nursing homes [33, 43].

We found higher comfort ratings in long-term care; the large UK VOICES study also found that care homes and hospices were rated better than home for non-cancer deaths, [7] and the Swedish NONA study found similar satisfaction with care amongst relatives of over-90-year-olds dying in care homes [80]. We recognise that an informant’s report of a comfortable death is a subjective measure that may be coloured by many factors and reported comfort in care homes may reflect family hopes or even staff defensiveness, but these conjectures are beyond what the data can elucidate. The non-significant association of moderate-severe dementia with reported comfort is somewhat supported by the VOICES study which found that relatives of people who died with dementia tended to rate the overall level of care better than ratings for those dying without dementia, though they were less likely to rate care excellent. [7] Authors of another study which found it paradoxical that end-of-life care was improved when the person had dementia hypothesised this was possibly due to increased attention levels [81].

The symptom burden is also similar to that found in other research, with pain and distress being common symptoms found in hospitals, care homes and at home in other studies [82–86], and inadequate symptom relief also reported as common [86]. There is discordance in the literature about the impact of symptoms on quality of dying with some finding their presence has no effect [84]; we also found no significant association between symptom presence and comfort.

It is heartening that these very old people, including those with dementia, were described as being comfortable at the end of life. However, the high symptom prevalence, some reportedly inadequately addressed, reinforces calls to improve end-of-life care from a plethora of recent reports [87–89]. That symptoms were common is relevant to current attention to the design and delivery of palliative care [90, 91], particularly the low proportion of depression treated. Mental health at the end of life for the very old needs to be assessed and addressed, likewise the adequacy of pain relief offered, especially considering the inter-play of dementia, depression and distress.

It is important to note the strong inter-relationship of many variables we considered. People with high support needs at home are least likely to ‘stay in place’. People with dementia at advanced ages are more likely to live in care homes and experience fewer end-of-life transitions to other settings. From a policy perspective the finding that long-term care settings are associated with dying comfortably supports recent evidence that is starting to encourage re-thinking the focus on dying at home [20, 22, 25, 26, 30, 92], particularly for this very old age group. Cost-savings arguments based on cancer care service models may be inappropriate to the very different palliative care context for frail older people [53, 55, 93], who cannot always be adequately supported at home [94–97]. There is growing recognition that good care homes increasingly play a role akin to hospices for the frail elderly [98, 99], but common public perceptions [100] tend more to reflect concerns over the wide variability in standards and the challenges many homes face in providing end-of-life care [101–104]. Recent research confirms this is no less a challenge at home [33, 48, 51, 105–110], the setting where the fewest very old people die, though our findings suggest home can be a comfortable place of death provided circumstances support staying there. However, the number of older old people dying in acute hospitals, where we found the odds of dying comfortably were lowest, makes this another priority area for improving end-of-life care [111–119].

Informants sometimes found it difficult to say when the “final illness” began, even in retrospect: this is not unexpected, given many participants’ health had been fragile long before death [120]. In all settings, recognising at what point along a frailty trajectory someone is dying is a challenge [49, 80, 104, 121–123] that sometimes hinders advance care planning (ACP). Older people and their families’ choices need to be fully informed through realistic discussion anticipating deterioration, something many, but not all, very old people would welcome [105, 124–127]. Early and more widespread discussion of place of care and death, using clear language and frank information could be helpful in reducing the discomfort in talking about death, alleviating some distress and opening communication channels for better symptom control. Transfer to a preferred place of care should be facilitated whenever possible, but all too commonly a patient is admitted to hospital without those concerned having considered where they can be best cared for, or would wish to die. If social/community care use is predictive of end-of-life comfort, as found in our study, this has implications that can guide planning future management of considerable care needs. However, although ACP may reduce unwanted transitions, including hospital admissions [17, 128], even well-informed choices discussed in advance are no guarantee when services cannot cope with changed circumstances [129]. This very unpredictability underlies some reluctance to make plans [105, 124, 127] and poses big questions for future research and practice: how care for the very old and frail at the end of their lives can be responsive to their preference and deliver the best possible support in all settings for dying comfortably [130].

## Conclusions

This study’s findings are consistent with reports that care homes can provide care akin to hospice for the very old and support an approach of supporting residents to stay in their care home or own home if possible. The reported high prevalence of multiple symptoms, but less frequently reported symptom control, can inform policy and training to improve older old people’s end-of-life care in all settings.

## Additional files

Additional file 1: Table S1. Factors potentially related to comfort during the final illness. Factors found in univariate logistic regression analyses to be significantly associated with reported comfort during the final illness related to the place of end-of-life care and death, to transitions between these and, for those living at home when last interviewed, to the level of support received from services. As many variables of interest were clearly inter-related, Table S1 shows the effects of adjusting for key significant variables separately on odds ratios associated with all factors potentially related to reported comfort (The ‘transitions at the end of life’ variable is derived from ‘place of care in the final illness’ and ‘place of death’). Sample size limitations precluded full stepwise multivariable regression modelling. Cognition was hypothesised a priori to be important so, although univariate analyses did not find this a significant factor, ORs adjusted for dementia/cognitive status are also shown: this adjustment slightly reduced effect sizes but did not remove significance of any associated factors. (DOC 131 kb)

Additional file 2: Table S2. Factors potentially related to comfort during the final illness – odds ratios adjusted for interview time intervals. Adjusting for the time interval between last study interviews and participants’ deaths, and between participants’ deaths and retrospective informant interviews after death, made minimal or no difference to associations between reported comfort in the final illness and other potentially related factors. (DOC 125 kb)

## Abbreviations

CAMDEX: Cambridge Mental Disorders of the Elderly Examination
CC75C: Cambridge City over-75s Cohort
CI: Confidence Interval
DSM-IV: Diagnostic and Statistical Manual of Mental Disorders version 4
GP: General practitioner
MMSE: Mini Mental State Examination
OR: Odds Ratio
UK: United Kingdom
VOICES: Views of Informal Carers - Evaluation of Services
