# Supplementary material for: Dying comfortably in very old age with or without dementia in different care settings – a representative “older old” population study
Source: BMC Geriatr. 2017 Oct 5;17:222. doi: 10.1186/s12877-017-0605-2 (PMC5628473; doi:10.1186/s12877-017-0605-2)
Supplement: Supplementary file 1 — Factors potentially related to comfort during the final illness. Factors found in univariate logistic regression analyses to be significantly associated with reported comfort during the final illness related to the place of end-of-life care and death, to transitions between these and, for those living at home when last interviewed, to the level of support received from services. As many variables of interest were clearly inter-related, Table S1 shows the effects of adjusting for key significant variables separately on odds ratios associated with all factors potentially related to reported comfort (The ‘transitions at the end of life’ variable is derived from ‘place of care in the final illness’ and ‘place of death’). Sample size limitations precluded full stepwise multivariable regression modelling. Cognition was hypothesised a priori to be important so, although univariate analyses did not find this a significant factor, ORs adjusted for dementia/cognitive status are also shown: this adjustment slightly reduced effect sizes but did not remove significance of any associated factors. (DOC 131 kb) [file 12877_2017_605_MOESM1_ESM.doc]

***Additional file 1: Table S1 Factors potentially related to comfort during the final illness (Supplemental file for web only data)***

|  | **n reported as “comfortable” in their final illness / total n**  **for each group**  **(%)** | **Unadjusted Odds Ratio (OR) for reported comfort**  **(95% C.I.)** | **OR adjusted for places of care and death / EoL transitions**  **(95% C.I.)** | **OR adjusted for dying**  **at usual address**  **(95% C.I.)** | **OR adjusted for receipt**  **of services (community only: n=134)**  **(95% C.I.)** | **OR adjusted for dementia**  **/ cognitive status**  **(95% C.I.)** |
| --- | --- | --- | --- | --- | --- | --- |
| **Socio-Demographics** |  |  |  |  |  |  |
| **Age** |  |  |  |  |  |  |
| < 90 years old | 62/78 (79) | 1 | 1 | 1 | 1 | 1 |
| ≥ 90 years old | 83/102 (81) | 1.1 (0.5 - 2.4) | 1.1 (0.5 - 2.4) | 0.9 (0.4 - 1.9) | 1.1 (0.5 - 2.5) | 1.0 (0.5 - 2.2) |
| **Sex** |  |  |  |  |  |  |
| Male | 46/57 (81) | 1 | 1 | 1 | 1 | 1 |
| Female | 99/123 (80) | 1.0 (0.4 - 2.2) | 0.6 (0.2 - 1.5) | 0.9 (0.4 - 2.0) | 1.0 (0.4 - 2.4) | 0.8 (0.4 - 1.9) |
| **Marital status** * |  |  |  |  |  |  |
| Married | 31/38 (82) | 1 | 1 | 1 | 1 | 1 |
| Widowed | 98/123 (80) | 0.9 (0.3 - 2.2) | 0.8 (0.3 - 2.4) | 0.8 (0.3 - 2.1) | 0.9 (0.3 - 2.8) | 1.0 (0.4 - 2.5) |
| Single | 14/17 (82) | 1.1 (0.2 - 4.7) | 1.0 (0.2 - 5.3) | 0.8 (0.2 - 4.0) | 1.0 (0.2 - 5.4) | 1.2 (0.3 - 5.3) |
| **School leaving age** * |  |  |  |  |  |  |
| ≤ 14 years old | 90/115 (78) | 1 | 1 | 1 | 1 | 1 |
| ≥ 15 years old | 54/64 (84) | 1.5 (0.7 - 3.4) | 1.2 (0.5 - 2.9) | 1.4 (0.6 - 3.2) | 1.5 (0.6 - 3.8) | 1.5 (0.7 - 3.5) |
| **Social class** *† |  |  |  |  |  |  |
| Non-manual | 61/79 (77) | 1 | 1 | 1 | 1 | 1 |
| Manual | 82/99 (83) | 1.4 (0.7 - 3.0) | 1.5 (0.7 - 3.5) | 1.6 (0.7 - 3.4) | 1.6 (0.7 - 3.8) | 1.6 (0.7 - 3.4) |
| **Place of residence**  **at last interview** ** |  |  |  |  |  |  |
| Home | 104/134 (78) | 1 | 1 | 1 | - | 1 |
| Long-term care | 41/46 (89) | 2.4 (0.9 - 6.5) | 1.1 (0.3 - 3.8) | 1.3 (0.3 - 3.9) | - | 1.5 (0.5 - 4.7) |
| */Table S1 cont.* | **n reported as “comfortable” in their final illness / total n**  **for each group**  **(%)** | **Unadjusted Odds Ratio (OR) for reported comfort**  **(95% C.I.)** | **OR adjusted for places of care and death / EoL transitions**  **(95% C.I.)** | **OR adjusted for dying**  **at usual address**  **(95% C.I.)** | **OR adjusted for receipt**  **of services (community only: n=134)**  **(95% C.I.)** | **OR adjusted for dementia**  **/ cognitive status**  **(95% C.I.)** |
| **Location of End of Life Care** |  |  |  |  |  |  |
| **Place of care during final illness** ** |  |  |  |  |  |  |
| Hospital | 55/75 (73) | 1 | 1 | 1 | 1 | 1 |
| Home | 24/33 (73) | 1.0 (0.4 - 2.4) | 2.4 (0.4-11.5) | 0.6 (0.2 - 1.6) | 1.0 (0.4 - 2.7) | 1.0 (0.4 - 2.4) |
| Long-term care | 66/72 (92) | **4.0 (1.5-10.7)** | **4.3 (1.5-12.5)** | 1.7 (0.5 - 5.7) | 3.6 (0.9-13.5) | **3.3 (1.1 - 9.9)** |
| **Place of death** ** |  |  |  |  |  |  |
| Hospital | 59/85 (69) | 1 | 1 | 1 | 1 | 1 |
| Home | 17/19 (89) | 3.7 (0.8-17.4) | 2.4 (0.4-11.5) | 1.6 (0.2-14.9) | 3.3 (0.7-16.0) | 3.8 (0.8-17.6) |
| Long-term care | 69/76 (91) | **4.3 (1.8-10.7)** | **4.3 (1.5-12.5)** | 2.5 (0.7 - 9.3) | **5.2 (1.4-19.3)** | **3.5 (1.3 - 9.5)** |
| **Places of care and death**  **- transitions at the end of life** *‡ |  |  |  |  |  |  |
| Care in final illness: hospital  Place of death: hospital | 53/71 (75) | 1 | - | 1 | 1 | 1 |
| Final illness: home  Place of death: hospital | 6/13 (46) | ***0.3 (0.1 - 1.0)*** | - | ***0.3 (0.1 - 1.0)*** | 0.4 (0.1 - 1.3) | ***0.3 (0.1 - 1.0)*** |
| Final illness: home  Place of death: home | 14/16 (88) | 2.4 (0.5-11.5) | - | 2.2 (0.1-35.0) | 2.2 (0.4-10.9) | 2.4 (0.5-11.7) |
| Final illness: long-term care  Place of death: long-term care | 64/69 (93) | **4.3 (1.5-12.5)** | - | 4.1 (0.5-33.6) | ***4.7 (1.0-22.5)*** | ***3.2 (1.0-10.3)*** |
| **Dying at usual address** |  |  |  |  |  |  |
| No | 76/105 (72) | 1 | 1 | - | 1 | 1 |
| Yes | 69/75 (92) | **4.4 (1.7-11.2)** | 1.1 (0.1-10.6) | - | **4.2 (1.2-15.1)** | **3.7 (1.4 - 9.7)** |

| */Table S1 cont.* | **n reported as “comfortable” in their final illness / total n**  **for each group**  **(%)** | **Unadjusted Odds Ratio (OR) for reported comfort**  **(95% C.I.)** | **OR adjusted for places of care and death / EoL transitions**  **(95% C.I.)** | **OR adjusted for dying**  **at usual address**  **(95% C.I.)** | **OR adjusted for receipt**  **of services (community only: n=134)**  **(95% C.I.)** | **OR adjusted for dementia**  **/ cognitive status**  **(95% C.I.)** |
| --- | --- | --- | --- | --- | --- | --- |
| **Health & Disability** |  |  |  |  |  |  |
| **Dementia status** |  |  |  |  |  |  |
| No dementia-cognitively intact | 25/33 (76) | 1 | 1 | 1 | 1 | - |
| Cognitively impaired +/- minimal/mild dementia | 61/81 (75) | 1.0 (0.4 - 2.5) | 1.0 (0.4 - 2.7) | 0.9 (0.3 - 2.3) | 0.9 (0.3 - 2.4) | - |
| Moderate/severe dementia | 59/66 (89) | 2.7 (0.9 - 8.2) | 1.9 (0.5 - 7.2) | 1.7 (0.5 - 5.4) | 2.6 (0.6-11.0) | - |
| **Duration of final illness** * |  |  |  |  |  |  |
| Less than a week | 39/44 (89) | 1 | 1 | 1 | 1 | 1 |
| 7 days up to a month | 62/80 (78) | 0.5 (0.2 - 1.4) | 0.6 (0.2 - 1.7) | 0.6 (0.2 - 1.7) | 0.5 (0.1 - 1.7) | 0.5 (0.2 - 1.4) |
| 1 month or more | 44/56 (79) | 0.5 (0.2 - 1.5) | 0.7 (0.2 - 2.3) | 0.6 (0.2 - 1.9) | 0.5 (0.1 - 1.9) | 0.5 (0.2 - 1.6) |
| **No. of hospital admissions /year since last interview** * |  |  |  |  |  |  |
| None | 79/98 (81) | 1 | 1 | 1 | 1 | 1 |
| One or more | 59/75 (79) | 0.9 (0.4 - 1.9) | 1.0 (0.5 - 2.4) | 1.0 (0.5 - 2.2) | 0.9 (0.4 - 2.2) | 0.9 (0.4 - 1.9) |
| **Functional disabilities in ADLs** |  |  |  |  |  |  |
| No ADL disability  (neither basic or instrumental) | 8/10 (80) | 1 | 1 | 1 | 1 | 1 |
| Instrumental ADL disability only | 14/ 20 (70) | 0.6 (0.1 - 3.6) | 0.5 (0.1 - 3.7) | 0.6 (0.1 - 3.9) | 0.8 (0.1 - 5.1) | 0.6 (0.1 - 4.0) |
| Basic + instrumental ADL disability | 123/150 (82) | 1.1 (0.2 - 5.7) | 0.9 (0.2 - 5.1) | 1.0 (0.2 - 4.9) | 1.9 (0.3-10.7) | 0.9 (0.2 - 4.8) |
| **Receiving service support (*excluding long-term care*)*** |  |  |  |  |  |  |
| More than once a week | 33/51 (65) | 1 | 1 | 1 | - | 1 |
| Once a week | 32/38 (84) | ***2.9 (1.0 - 8.3)*** | 2.4 (0.8 - 7.3) | 2.6 (0.9 - 7.9) | - | ***2.7 (1.0 - 7.9)*** |
| None | 35/41 (85) | **3.2 (1.1 - 9.0)** | 2.7 (0.9 - 8.3) | 2.7 (0.9 - 8.0) | - | **3.0 (1.1 - 8.6)** |

| */Table S1 cont.* | **n reported as “comfortable” in their final illness / total n**  **for each group**  **(%)** | **Unadjusted Odds Ratio (OR) for reported comfort**  **(95% C.I.)** | **OR adjusted for places of care and death / EoL transitions**  **(95% C.I.)** | **OR adjusted for dying**  **at usual address**  **(95% C.I.)** | **OR adjusted for receipt**  **of services (community only: n=134)**  **(95% C.I.)** | **OR adjusted for dementia**  **/ cognitive status**  **(95% C.I.)** |
| --- | --- | --- | --- | --- | --- | --- |
| **Informants & Interviews** |  |  |  |  |  |  |
| **Informant’s sex** |  |  |  |  |  |  |
| Male | 50/57 (88) | 1 | 1 | 1 | 1 | 1 |
| Female | 95/123 (77) | 0.5 (0.2 - 1.2) | 0.5 (0.2 - 1.4) | 0.5 (0.2 - 1.1) | **0.2 (0.1 - 0.6)** | 0.5 (0.2 - 1.1) |
| **Relationship to participant** * |  |  |  |  |  |  |
| Husband or wife | 15/18 (83) | 1 | 1 | 1 | 1 | 1 |
| Son or daughter | 78/97 (80) | 0.8 (0.2 - 3.1) | 0.6 (0.2 - 2.9) | 0.7 (0.2 - 2.7) | 1.0 (0.2 - 4.4) | 0.8 (0.2 - 3.2) |
| Other relative | 8/12 (67) | 0.4 (0.1 - 2.2) | 0.3 (<0.1-2.1) | 0.3 (0.1 - 1.8) | 0.5 (0.1 - 3.4) | 0.4 (0.1 - 2.4) |
| Friend | 6/8 (75) | 0.6 (0.1 - 4.5) | 0.4 (<0.1-3.8) | 0.5 (0.1 - 3.9) | 0.3 (<0.1-3.1) | 0.6 (0.1 - 4.9) |
| Warden or matron | 13/15 (87) | 1.3 (0.2 - 9.0) | 0.8 (0.1 - 8.6) | 0.7 (0.1 - 5.3) |  | 0.8 (0.1 - 6.0) |
| Other | 23/28 (82) | 0.9 (0.2 - 4.4) | 0.6 (0.1 - 4.1) | 0.6 (0.1 - 2.9) | 1.2 (0.2 - 7.4) | 0.9 (0.2 - 4.3) |
| **How often saw participant** |  |  |  |  |  |  |
| Lived with participant | 24/29 (83) | 1 | 1 | 1 | 1 | 1 |
| Daily | 35/45 (78) | 0.7 (0.2 - 2.4) | 0.4 (0.1 - 1.6) | 0.6 (0.2 - 2.0) | 1.3 (0.3 - 5.1) | 0.6 (0.2 - 2.0) |
| More than once a week | 45/55 (82) | 0.9 (0.3 - 3.1) | 0.4 (0.1 - 1.7) | 0.7 (0.2 - 2.2) | 0.9 (0.2 - 3.2) | 0.8 (0.2 - 2.7) |
| Once a week | 18/22 (82) | 0.9 (0.2 - 4.0) | 0.7 (0.1 - 3.8) | 0.7 (0.2 - 3.2) | 1.6 (0.3 - 9.1) | 0.8 (0.2 - 3.7) |
| Less than once a week | 23/29 (79) | 0.8 (0.2 - 3.0) | 0.5 (0.1 - 2.4) | 0.7 (0.2 - 2.7) | 0.9 (0.2 - 4.0) | 0.7 (0.2 - 2.9) |
| **Interval from last survey interview to death** |  |  |  |  |  |  |
| < median | 73/90 (81) | 1 | 1 | 1 | 1 | 1 |
| ≥ median | 72/90 (80) | 0.9 (0.4 - 1.9) | 1.1 (0.5 - 2.6) | 1.0 (0.5 - 2.1) | 1.0 (0.4 - 2.4) | 0.8 (0.4 - 1.8) |
| **Interval from death**  **to informant interview** |  |  |  |  |  |  |
| < median | 70/90 (78) | 1 | 1 | 1 | 1 | 1 |
| ≥ median | 75/90 (83) | 1.4 (0.7 - 3.0) | 1.0 (0.4 - 2.3) | 1.4 (0.6 - 2.9) | 1.8 (0.8 - 4.3) | 1.5 (0.7 - 3.3) |

***Footnotes***

OR = Odds Ratio (shown to 1 decimal point)

**bold OR** = significant (p<0.05); ***italic bold OR*** = borderline significant (p=0.5)

95% C.I. = 95% Confidence Interval (shown to 1 decimal point)

EoL = end of life

ADL(s) = Activity (*or* Activities) of Daily Living

* Variables in which categories total <180 had missing data.

† Social class categorised following contemporary UK Office of National Statistics grading of occupation reported at baseline interview:

Non-manual = I, II or IIIa, Manual = IIIb, IV or V

** Home: community-dwelling in a house, flat, ‘granny flat’ (part of a relative’s home) or sheltered accommodation

Long-term care: in an older people’s residential home, nursing home or long-stay ward

‡ To explore the effects of transitions in place of care at the very end of life, the variable *Places of care at the end of life* was derived from data on where each individual

was cared for during their final illness and where they died. Frequencies in other categories were too small (between 1 and 4 people) to calculate any estimates for

other combinations of place of care.

**Table S1 legend:**

Factors found in univariate logistic regression analyses to be significantly associated with reported comfort during the final illness related to the place of end-of-life care and death, to transitions between these and, for those living in the community when last interviewed, to the level of support received from services. As many variables of interest were clearly inter-related, Table S1 shows the effects of adjusting for key significant variables separately on odds ratios associated with all factors potentially related to reported comfort (The ‘transitions at the end of life’ variable is derived from ‘place of care in the final illness’ and ‘place of death’). Sample size limitations precluded full stepwise multivariable regression modelling. Cognition was hypothesised a priori to be important so, although univariate analyses did not find this a significant factor, ORs adjusted for dementia/cognitive status are also shown: this adjustment slightly reduced effect sizes but did not remove significance of any associated factors.
